# Prevalence of obesity among Bangladeshi pregnant women at their first trimester of pregnancy

**DOI:** 10.5195/cajgh.2013.70

**Published:** 2013-11-18

**Authors:** Shatabdi Goon

**Affiliations:** Nutrition and Food Engineering Department, Daffodil International University, Dhanmondi, Dhaka, Bangladesh

**Keywords:** obese pregnant, first trimester, adverse pregnancy outcome, body mass index, neonatal health, maternal health

## Abstract

**BACKGROUND:**

Paradoxically, the escalating global epidemic of maternal obesity coexists with malnutrion in many areas of Bangladesh. This proves a major challenge to obstetric practice from preconception to postpartum due to related comorbid conditions including: maternal death or severe morbidity, gestational diabetes and hypertension, increased risk of early and recurrent miscarriage, pre-eclampsia, thromboembolism, post-caesarean wound infection, postpartum haemorrhage, and low breastfeeding rates. A dramatic increase in birth defects and other pregnancy-induced disorders related to maternal obesity has added millions of dollars to health care costs leading great economical loss to the country.

**OBJECTIVE:**

The study was designed to determine the prevalence of obesity among Bangladeshi pregnant women in their 1^st^ trimester of pregnancy.

**STUDY DESIGN:**

426 pregnant women presenting to the antenatal care until of Azimpur maternity hospital of Dhaka, Bangladesh were randomly selected for this cross sectional study to determine their weight status using body mass index (BMI, kg/m^2^).

**RESULT:**

90 (21.2%) pregnant women were reported as obese with pregnancy BMI of >30 kg/m^2^. 171 (40.1%) and 140 (32.8%) pregnant women were reported as overweight and healthy with pregnancy BMI of 25–29.9 kg/m^2^ and 18.5–24.9 kg/ m^2^, respectively. Statistical analysis revealed obesity and overweight status were found to be significantly associated with age; women aged 31 or above were more likely to be obese (OR=2.5; 95% CI 1.53–3.96) and overweight (OR=3.3; 95% CI 2.15–4.99).

**CONCLUSION:**

This study provides evidence of increasing trends in obesity among Bangladeshi pregnant women, which poses possible health risks both for mother and child. The findings of this study may act as baseline data for monitoring the effectiveness of national programs for the prevention and control of maternal obesity.

Maternal obesity and related comorbid conditions have serious impact on the health and development of obese women’s offspring. The incidence of maternal obesity at the start of pregnancy is increasing worldwide. 1 International studies show a prevalence of maternal obesity ranging from 1.8% to 25.3% across countries. 2 Approximately 50% of pregnant women have a body mass index (BMI) >25 kg/m.^2^,^3^ Nearly two-thirds of reproductive-age women in the United States (U.S.) are currently overweight or obese, placing them at elevated risk for adverse health outcomes. 4 The recent National Health and Nutrition Examination Survey (NHANES) found that in the United States, more than 50% of pregnant women are overweight or obese and 8% of reproductive-aged women are extremely obese. 5 In 2009, 26% of adult, reproductive-age, U.S. women were classified with a BMI ≥30 kg/m^2^. 6

Compared to developed countries, maternal obesity is less of an epidemic in developing ones; however, Bangladeshi women of reproductive age have shown a trend of increasing BMI. 7 A survey conducted among this subpopulation found obesity prevalence increased from 2.7% to 8.9% between 1996 and 2006. 7 In comparison, the prevalence of maternal obesity in the United States ranged from 13.9% to 25.1% between 2004 and 2005. 8 Current Institute of Medicine guidelines, published in 1990, recommend that all women can expect to gain one or two kilograms in their first trimester of pregnancy, but additional weight gain above is considered excessive. 9 In recent years, excessive weight gain has led to increased obesity prevalence among pregnant women, resulting in maternal and fetal health complications. Maternal obesity carries significant risks for the mother and fetus with increased health risks to the mother during the antenatal, intrapartum, and postnatal periods. 10–13 Excess accumulation of adipose tissue within the abdominal cavity, or visceral obesity, among obese, pregnant women has been associated with a cluster of metabolic alterations, which includes: insulin resistance, hyperinsulinemia, elevated triglyceride levels, low HDL cholesterol, and hypertension. 14,15 Evidence from both animal and human studies indicates that maternal obesity 1) increases the risk for offspring to develop obesity, 2) alters body composition in child- and adulthood, and 3) impacts the offspring’s cardio-metabolic health with dysregulation of metabolism, including: insulin homoeostasis, development of hypertension, and vascular dysfunction. 16

Maternal obesity is associated with increased odds of neural tube defects, 17–22 spina bifida, 17 cardiovascular anomalies, 23 and high mortality rate. Reynolds *et al.* found that offspring of obese mothers have an increased risk of hospital admission for a cardiovascular event (OR=1.29; 95% CI 1.06–1.57) compared with offspring of mothers with normal BMI. 24 First trimester maternal obesity has significantly increased over time, having more than doubled from 7.6% to 15.6% since 1994, 25 resulting in lost pregancies and birth defects 26 that occur during embryogenesis. Overweight and obese women have larger anatomic depots of adipose tissue in all compartments, resulting in different metabolic adaptations. 27 Obese women are more likely to develop gestational diabetes, 28,29 high blood pressure, 30–33 and proteinuria 34–37 after 20 weeks of pregnancy, which promote preeclampsia. 38,39 Compared with normal weight women (BMI < 25 kg/m^2^), a recent meta-analysis of 20 studies demonstrated that the OR of developing gestational diabetes was 2.14 (95% CI 1.82–2.53), 3.56 (95% CI 3.05–4.21), and 8.56 (95% CI 5.07–16.04) among overweight (BMI: 25–30 kg/m^2^), obese (BMI > 30 kg/m^2^), and severely obese women (BMI > 40 kg/m^2^), respectively. 29 Obesity among pregnant women has also been linked to poorer cognitive performance, higher incidence of autism spectrum disorders, and more attention deficit-hyperactivity disorder (ADHD) in their children. Increased BMI is associated with increased rates of midline vertical incision, longer operative time, urinary tract infections, 40 stillbirths and fetal death, 41–44 lower rates of subcuticular skin closure, 45 and caesarean delivery. 46 A meta-analysis of 33 studies showed that the odd ratio (OR) of cesarean delivery were 1.46 (95% CI 1.34–1.60), 2.05 (95% CI 1.86–2.27), and 2.89 (95% CI 2.28–3.79) among overweight, obese, and severely obese women, respectively, compared with normal weight pregnant women. 10,11,47,48 Also in a recent meta-analysis, a BMI ≥ 25 was found to be associated with miscarriage, regardless of mode of conception (OR=1.67; 95% CI 1.25–2.25). 49 Obesity increases the risk of preterm delivery, 50 (a leading cause of infant mortality, morbidity, and long-term disability). 51 These risks increase with decreasing gestational age. 52 Obese women are at increased risk of thrombosis as well as delivering an infant significantly larger than average (macrosomia). 46,53,54

Evidence shows that a child of an obese mother may suffer from exposure to a suboptimal in utero environment and that early life adversities may extend into adulthood. 55 Being obese during pregnancy might increase the risk that the baby will develop heart disease or diabetes as an adult. Research suggests that obesity during pregnancy slightly increases the risk of having a baby who’s born with a birth defect, 3 such as heart problems or conditions affecting the brain or spinal cord. 19 Higher maternal BMI at first prenatal hospital visit is associated with increased risk of prolonged pregnancy and increased rate of induction of labor. 56

Maternal obesity offers an altered genetic, hormonal, and biochemical environment for the developing fetus/embryo and influences fetal growth and organ development. 57 Compared with neonates born to women of normal weight, neonates born to women with BMIs ≥40 (severely obese) were at increased risk of birth injury to the peripheral nervous system, birth injury to the skeleton, respiratory distress syndrome, bacterial sepsis, convulsions, and hypoglycemia. 58 Increased maternal BMI is associated with categorical and continuous reductions in the proliferative index and a continuous reduction in the apoptotic index. 59 Obese women are also less likely to initiate and sustain breastfeeding. 60 Women who are obese during pregnancy might be at increased risk of a potentially serious sleep disorder in which breathing repeatedly stops and starts. Sleep apnea occurring in this group of women may further complicate anesthetic management and postoperative care. 61

Helping women understand the risks associated with obesity and working with them to develop strategies to decrease their risk is a challenge for both the patient and the healthcare provider. The objective of this study was to evaluate the prevalence of maternal obesity among Bangladeshi pregnant women.

## Methods

### Study design

This cross-sectional study was carried out at Azimpur maternity hospital, Dhaka, Bangladesh between May and June 2013. A total of 450 urban, pregnant women in their first trimester of pregnancy were selected by systemic random process by approaching every 2^nd^ woman attending to prenatal care clinic. After approaching potential participants, 426 pregnant women (94.6%) agreed to participate. 24 pregnant women refused consent. Verbal, informed consent was obtained from all study subjects per the Bangladesh Medical Research Council.

### Inclusion/ Exclusion Criteria

Women were approached to participate in this investigation if they were currently in their first trimester of pregnancy and attending the prenatal care clinic of Azimpur maternity hospital. Women were exluded from analysis if they had a history of hypertension, diabetes, or spontaneous abortions. Thus, 426 pregnant women were enrolled and included in analysis.

### Data Collection

Participants completed questionnaires providing information about age, month of pregnancy, and education status. Age was stratified into 4-year categories: <20 years, 21–25 years, 26–30 years, and >30 years.

All demographic information was collected via face-to-face interview. Body mass index (BMI) (kg/m^2^) was calculated based on clinically assessed weight (kg) and height (m) at baseline. The current analysis considered only the baseline measurements, as there was no notable change with regard to BMI during the follow up across the trials. WHO definitions were used to categorize women as normal weight (BMI: 18.5–24.9), overweight (BMI: 25–29.9), and obese (BMI: 30+). Substantial weight gain typically does not occur during the first trimester of pregnancy, therefore anthropometric measurements taken at baseline were compared to prepregnancy measurements found in the patients’ medical records. No significant differences were found between the two measurements therefore no patients were excluded. Weight and height were measured by pre-defined procedure using a standard weight scale and measuring tape.

### Statistical analysis

Considering an expected obesity prevalence of 50% and using a confidence level of 95% the sample size of the cross-sectional study was calculated as 450 using the following formula:

$$\text{n}=\frac{{\text{t}}^{2}*\text{p}(1-\text{p})}{{\text{m}}^{2}}$$

Data were analyzed using IBM SPSS^®^ statistics version 15.0. 62 Descriptive statistics were used for demographic information and Chi-squared tests were used to test significance of associations between age and BMI.

## Results

This paper presents the weight status (determined by BMI) in a sample of Bangladeshi pregnant women at their first trimester of pregnancy. 426 women were interviewed and examined. The mean age of the selected pregnant women was 27.13 ± 5.38(Mean ± SD). 66 (15.5%) pregnant women were <20 years of age, 114 (26.8%) 21–25 years of age. 108 (25.4%), 26–30 years of age, and 138 (32.3%) were >30 years. 90 (21.2%) and 171 (40.1%) pregnant women were classified as obese or overweight with pregnancy. 140 (32.8%) pregnant women were normal weight. Table 1 shows the distribution of this sample by age and BMI level.

Figure one illustrates the prevalence of overweight (40.1%) and obese (21.2%) participants in this sample.

Among obese women, 48.9% were aged 31 or above. Additionally, approximately 48% of overweight women were aged of 31 or above. Women aged 31 years or above were more likely to be obese (OR=2.5; 95% CI 1.53–3.96) and overweight (OR=3.3; 95% CI 2.15–4.99). The correlation between age and BMI level was found to be significant, as shown in Table 2 (*p*=0.01).

Obesity was detected among 21.2% of participants. Those aged 31 years or above showed significantly higher prevalence (X^2^=14.18, *p*<0.05). Overweight was detected among 40.1% of participants and those aged 31 years or above showed significantly higher prevalence (X^2^=31.58, *p*<0.05). Table 3 illustrates the distribution of respondents according to weight status.

## Discussion

We determined the prevalence of obesity among a sample of 426 pregnant, Bangladeshi women who visited Azimpur maternity hospital in Dhaka, Bangladesh between May and June 2013. Approximately 40% of pregnant women were identified as overweight in their first trimester of pregnancy, whereas 21.2% were identified as obese. These findings mirror those of other literature. For example, Fattah *et al.* showed an obesity prevalence of 19% in a study of 1,000 Caucasian pregnant women. 63 Another study, conducted on pregnant women receiving maternal care in Bangladesh, showed an obesity prevalence of 23%. 64 Furthermore, A retrospective cohort study including 8,176 pregnant women showed an obesity prevalence of 17.7%, 35 Lastly, a cohort study of 4,830 patients with gestational diabetes (GDM) showed an obesity prevalence of 15.7%, 65 Flegal *et al.* showed the prevalence of obesity among US women of 35.8% in 2009–2010. 5 While the Centre for Maternal and Child Enquiries published that more than 1 in 20 pregnant women in US are severely obese, 66 this is one of the first studies that has been conducted in Bangladesh to evaluate the current data on maternal obesity.

In most cases, Bangladeshi women are considered malnourished during their gestational period, but this study has revealed pregnant, Bangladeshi women have relatively high prevalence of overweightness and obesity. Though this study was conducted only among Bangladeshi urban women, weight status of rural pregnant women could be different considering socio-demographical condition. As a developing country, Bangladesh has struggled with the burden of malnutrition; however, these new findings of obesity prevalence among pregnant Bangladesh women point to the possibility that obesity point to the possibility of obesity becoming an increasing problem to the Bangladeshi healthcare system. Previously, pregnant, Bangladeshi women typically participated in moderate household work, but with the extension of civilization, women are becoming dependent on modern technologies to cope with their daily work needs, decreasing their amount of daily, physical activity. As a result, obesity has more prevalent among this group. Economic, technologic, and lifestyle changes have created an abundance of cheap, high-calorie food coupled with a decrease in required physical activity, promoting excessive weight gain among this group.

Research suggests that as age increases, hormonal changes and decreased physical activity increases the risk of obesity. In this study, obesity was more prevalent among women aged 31 or more. Approximately 48.9% of obesity cases were found in women aged 31 years or higher. Overweightness was also higher among women aged 31 years or higher (48% of the sample). Other investigators showed that BMI increases with increasing maternal age. 50 Our study corroborates these findings, with a positive correlation between maternal age and BMI status (*p*=0.01). The risks of obesity in the first trimester are primarly pregnancy loss and birthday defects that occur during embryogenesis. Studies show that the risks of early miscarriage, and recurrent early miscarriage were significantly higher among obese patients. 67 The evidence is fairly clear that obesity is significantly associated with increased risk of pregnancy complications, ranging from miscarriage to problems later in pregnancy. Studies in genetically identical rodents convincingly show that maternal obesity, as well as elements of a hypercaloric diet, can permanently influence offspring risk of obesity, and these findings are also supported by studies in larger mammals. 68 Our findings highlight overweight and obesity as an important public health issue.

This study had several limitations. First, we only evaluated the weight status of Bangladeshi pregnant women corresponding to find out the prevalence of maternal obesity, not the maternal and fetal outcome associated with the prevalence of obesity among those pregnant women. Unless evaluating the pregnancy outcome, it will not be able to determine the overall risk associated with maternal obesity.

Considering this issue, the next step of this study will be to follow up respondents up to their delivery, and further research should evaluate the adverse pregnancy outcome associated with the maternal obesity. Second, data other than anthropometric measurements were self-reported, and the study is cross-sectional, which does not infer causal relationships. Lastly, we examined only one maternity hospital located in Dhaka, Bangladesh, so caution should be taken to generalize the data for other maternity hospitals and locations.

## Conclusion

The current study presented evidence for high rates of obesity among pregnant, Bangladeshi women in their first trimester of pregnancy. Babies born to obese mothers are more likely to be obese, develop diabetes, and have high blood pressure later in life, indicating that this issue not only affects the current generation, but may have long term implications in terms of quality of life and healthcare costs, among others. New strategies are needed to re-formulate existing obesity prevention and treatment programs by incorporating information on a healthy diet and lifestyle to produce holistic health and wellness interventions. Considering the high prevalence of obesity in females of reproductive age group in Bangladesh, this study emphasizes a strong need for pre-pregnancy advice about weight loss in these women before conception as even minimal weight loss can lead to better perinatal outcomes.

## Figures and Tables

**Figure 1:**
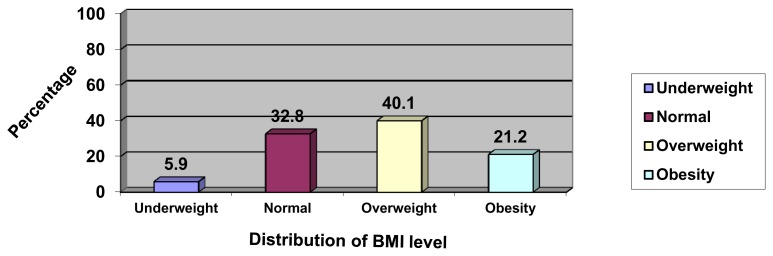
Distribution of respondents by BMI level.

**Table 1:** Distribution of respondents by age and BMI level.

Age	Total	Under weight	Normal (Healthy weight)	Overweight	Obesity
	426	25(5.9%)	140(32.8%)	171(40.1%)	90(21.2%)
**<20**	66(15.5%)	25(100%)	24(17.1%)	10(5.8%)	7(7.8%)
**21–25**	114(26.8%)	0	79(56.4%)	23(13.5%)	12(13.3%)
**26–30**	108(25.4%)	0	25(17.8%)	56(32.7%)	27(30%)
**≥31**	138(32.3%)	0	12(8.7%)	82(48%)	44(48.9%)

**Table 2:** Unadjusted OR for selected age (≥30) by BMI category.

Total	Overweight (BMI 25–29.9 kg/m^2^)		Obese (BMI >30 kg/m2)	
		OR(95% CI)		OR(95% CI)
261	n=171	3.3; 2.15–4.99	n=90	2.5 ;1.53–3.96

**Table 3:** Distribution of respondents according to the presence or absence of specific weight status.

Character	Present/absent	<20 years	21–25	26–30	≥31	Statistical values
**Obesity**	Present	7 (7.8%)	12 (13.3%)	27 (30%)	44 (48.9%)	X^2^=14.18, *p*<0.05
	Absent	59 (17.5%)	102 (30.4%)	81 (24.1%)	94 (28%)	
**Overweight**	Present	10 (5.8%)	23 (13.5%)	56 (32.7%)	82 (48%)	X^2^=31.58, *p*<0.05
	Absent	56 (21.9%)	91 (35.7%)	52 (20.4%)	56 (22%)	
**Normal weight**	Present	24 (17.1%)	79 (56.4%)	25 (17.9%)	12 (8.6%)	X^2^=54.03, *p*<0.05
	Absent	42 (14.7%)	35 (12.2%)	83 (29.0%)	126 (44.1%)	
**Underweight**	Present	25 (100%)	0	0	0	X^2^=12.73, *p*<0.05
	Absent	0	117 (29.2%)	108 (26.9%)	138 (43.9%)	
